# Coronal tibial slope is associated with accelerated knee osteoarthritis: data from the Osteoarthritis Initiative

**DOI:** 10.1186/s12891-016-1158-9

**Published:** 2016-07-19

**Authors:** Jeffrey B. Driban, Alina C. Stout, Jeffrey Duryea, Grace H. Lo, William F. Harvey, Lori Lyn Price, Robert J. Ward, Charles B. Eaton, Mary F. Barbe, Bing Lu, Timothy E. McAlindon

**Affiliations:** Division of Rheumatology, Tufts Medical Center, 800 Washington Street, Box #406, Boston, MA 02111 USA; Department of Radiology, Brigham & Women’s Hospital and Harvard Medical School, 75 Francis Street, Boston, MA 02115 USA; Medical Care Line and Research Care Line, Houston Health Services Research and Development (HSR&D) Center of Excellence Michael E. DeBakey VAMC, 2002 Holcombe Blvd, Houston, TX 77030 USA; Section of Immunology, Allergy, and Rheumatology, Baylor College of Medicine, 1 Baylor Plaza, BCM-285, Houston, TX 77030 USA; The Institute for Clinical Research and Health Policy Studies, Tufts Medical Center, Boston, MA 02111 USA; Tufts Clinical and Translational Science Institute, Tufts University, 800 Washington Street, Box #63, Boston, MA 02111 USA; Department of Radiology, Tufts Medical Center, 800 Washington Street, Box #299, Boston, MA 02111 USA; Center for Primary Care and Prevention, Alpert Medical School of Brown University, 111 Brewster St, Pawtucket, RI 02860 USA; Department of Anatomy and Cell Biology, Temple University School of Medicine, 3500 North Broad Street, Philadelphia, PA 19140 USA; Division of Rheumatology, Immunology & Allergy, Brigham & Women’s Hospital and Harvard Medical School, 75 Francis Street, Boston, MA 02115 USA

**Keywords:** Knee, Osteoarthritis, Bone, Alignment, Radiography

## Abstract

**Background:**

Accelerated knee osteoarthritis may be a unique subset of knee osteoarthritis, which is associated with greater knee pain and disability. Identifying risk factors for accelerated knee osteoarthritis is vital to recognizing people who will develop accelerated knee osteoarthritis and initiating early interventions. The geometry of an articular surface (e.g., coronal tibial slope), which is a determinant of altered joint biomechanics, may be an important risk factor for incident accelerated knee osteoarthritis. We aimed to determine if baseline coronal tibial slope is associated with incident accelerated knee osteoarthritis or common knee osteoarthritis.

**Methods:**

We conducted a case–control study using data and images from baseline and the first 4 years of follow-up in the Osteoarthritis Initiative. We included three groups: 1) individuals with incident accelerated knee osteoarthritis, 2) individuals with common knee osteoarthritis progression, and 3) a control group with no knee osteoarthritis at any time. We did 1:1:1 matching for the 3 groups based on sex. Weight-bearing, fixed flexion posterior-anterior knee radiographs were obtained at each visit. One reader manually measured baseline coronal tibial slope on the radiographs. Baseline femorotibial angle was measured on the radiographs using a semi-automated program. To assess the relationship between slope (predictor) and incident accelerated knee osteoarthritis or common knee osteoarthritis (outcomes) compared with no knee osteoarthritis (reference outcome), we performed multinomial logistic regression analyses adjusted for sex.

**Results:**

The mean baseline slope for incident accelerated knee osteoarthritis, common knee osteoarthritis, and no knee osteoarthritis were 3.1(2.0), 2.7(2.1), and 2.6(1.9); respectively. A greater slope was associated with an increased risk of incident accelerated knee osteoarthritis (OR = 1.15 per degree, 95 % CI = 1.01 to 1.32) but not common knee osteoarthritis (OR = 1.04, 95 % CI = 0.91 to 1.19). These findings were similar when adjusted for recent injury. Among knees with varus malalignment a greater slope increases the odds of incident accelerated knee osteoarthritis; there is no significant relationship between slope and incident accelerated knee osteoarthritis among knees with normal alignment.

**Conclusions:**

Coronal tibial slope, particularly among knees with malalignment, may be an important risk factor for incident accelerated knee osteoarthritis.

## Background

Accelerated knee osteoarthritis may be a unique subset of knee osteoarthritis, which develops definite osteophytes and joint space narrowing in less than 4 years (Kellgren-Lawrence [KL] Grade 0–1 to 3–4) [1–3]. Individuals who develop accelerated knee osteoarthritis are often older, overweight, and more likely to have a history of a recent knee injury than those with a slower onset of osteoarthritis or no osteoarthritis at all [1, 3]. Furthermore, individuals with accelerated knee osteoarthritis have greater knee pain and disability compared with those with a slower onset of knee osteoarthritis [4]. It is vital to identify risk factors for accelerated knee osteoarthritis to recognize people who will develop accelerated knee osteoarthritis and to identify early interventions.

**Fig. 1:**
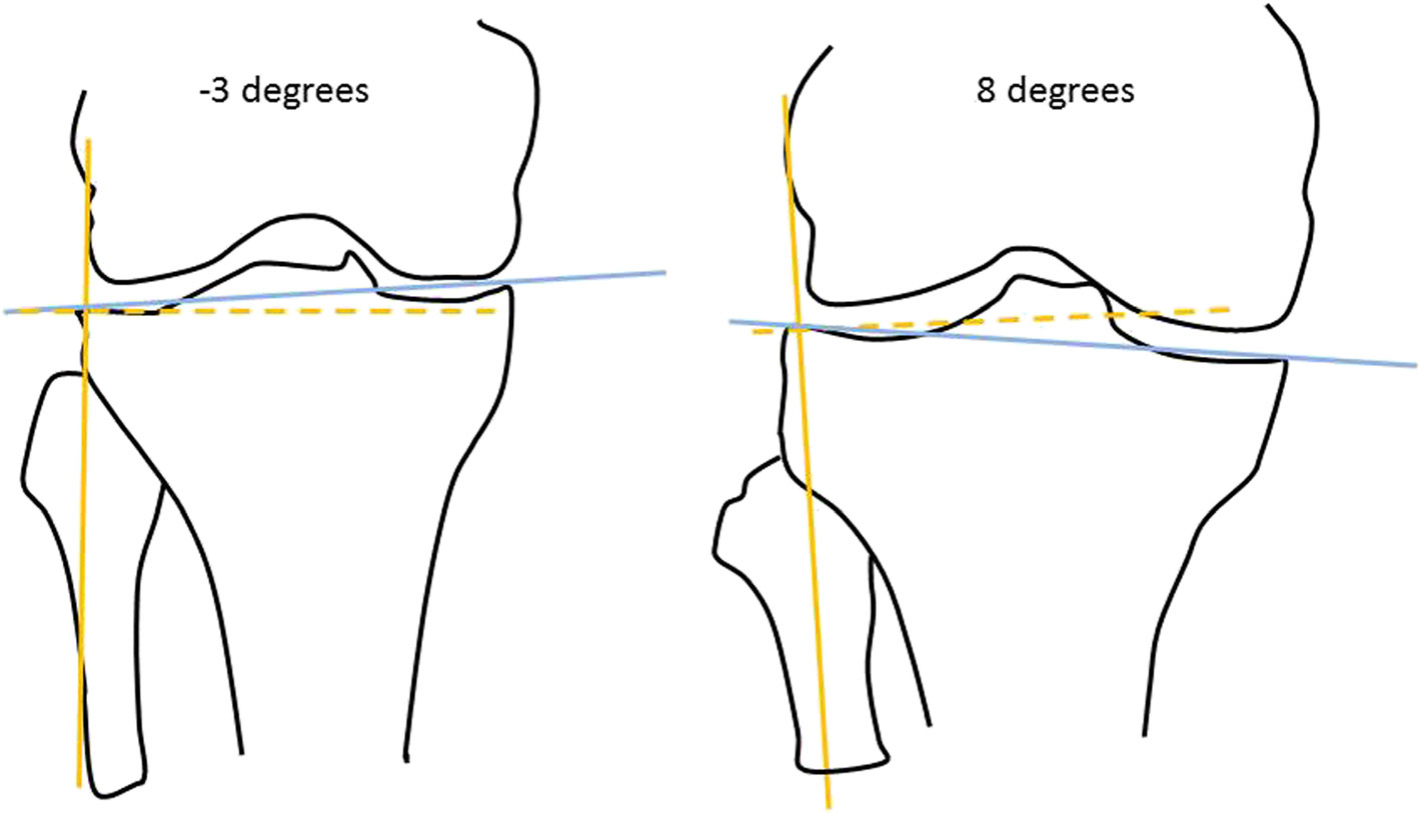
Examples of a positive and negative coronal tibial slope. The blue line connects the medial and lateral edges of the tibial plateau. The solid orange line is parallel to the longitudinal axis of the tibia and the dashed orange line is perpendicular to the longitudinal axis. A negative slope indicates that the lateral edge is lower than the medial edge of the plateau. A positive slope indicates that the lateral edge is more proximal than the medial edge of the plateau

The geometry of an articular surface (e.g., tibial slope), which is a determinant of altered joint biomechanics, may be an important risk factor for incident accelerated knee osteoarthritis. For example, coronal tibial slope, which is defined by the slope from the lateral edge of the tibial plateau to the medial edge (see Fig. 1), is associated with abnormal lower extremity biomechanics [5]. A positive slope represents a more proximal lateral plateau than medial. A greater coronal tibial slope is associated with greater knee internal rotation excursions and a lower coronal tibial slope is associated with greater knee valgus angles during a landing task [5]. Hence, an altered coronal slope angle may play a role in joint loading and influence the risk of injury. Due to these abnormal biomechanics, it is important to consider the coronal tibial slope as a potential risk factor for incident accelerated knee osteoarthritis. No prior study has evaluated coronal tibial slope in relation to osteoarthritis.

We aimed to determine if baseline coronal tibial slope is associated with incident accelerated knee osteoarthritis or a slower onset of knee osteoarthritis (common knee osteoarthritis) over 4 years. We hypothesized that individuals with greater or smaller coronal tibial slope angles will be more likely to develop accelerated or common knee osteoarthritis than those with a neutral coronal tibial slope. Furthermore, we explored a hypothesis that the coronal tibial slope will have a stronger association with incident accelerated knee osteoarthritis and common knee osteoarthritis among knees with malalignment. The goal of this work is to identify risk factors for incident accelerated knee osteoarthritis that may help us identify patients who need to be carefully monitored for the accelerated onset of knee osteoarthritis.

## Methods

To assess the association between coronal tibial slope and incident accelerated knee osteoarthritis or common knee osteoarthritis we conducted a case–control study using data and images from baseline and the first four years of follow-up in the Osteoarthritis Initiative (OAI). The OAI is a longitudinal observational study of adults with or at risk for knee osteoarthritis at four clinical sites in the United States: Memorial Hospital of Rhode Island, The Ohio State University, University of Maryland and Johns Hopkins University, and the University of Pittsburgh. The staff at the sites enrolled 4,796 men and women (45 to 79 years of age) between February 2004 and May 2006. Detailed descriptions of the eligibility criteria and the OAI protocol are publicly available at the OAI website [6]. This study received ethical approval from each OAI clinical site (Memorial Hospital of Rhode Island Institutional Review Board, The Ohio State University’s Biomedical Sciences Institutional Review Board, University of Pittsburgh Institutional Review Board, and University of Maryland Baltimore – Institutional Review Board), and the OAI coordinating center (Committee on Human Research at University of California, San Francisco). All participants provided informed consent to the OAI Study.

### Case and control definitions

We evaluated 3 groups that we defined based on radiographic definitions of knee osteoarthritis. The first group of cases was individuals with incident accelerated knee osteoarthritis, which we defined as someone with at least 1 knee that had no radiographic osteoarthritis at baseline (Kellgren-Lawrence [KL] grade < 2) that then developed advanced-stage knee osteoarthritis (KL grade 3 or 4, development of a definite osteophyte and joint space narrowing) within 48 months (*n* = 125). Among individuals with incident accelerated knee osteoarthritis, 54 (43 %) participants had no radiographic knee osteoarthritis in both knees at baseline. The second group of cases was individuals with common knee osteoarthritis progression who had no radiographic knee osteoarthritis (KL < 2) in both knees at baseline and at least 1 knee that increased in radiographic scoring within 48 months (excluding those with incident accelerated knee osteoarthritis; *n* = 187). The control group was individuals with no knee osteoarthritis who had no radiographic knee osteoarthritis at baseline in both knees and no change in KL grade in either knee from baseline to 48-month follow-up (*n* = 1,325).

To ensure timely completion of imaging assessments, we did 1:1:1 matching for the three groups based on sex. Matching was completed at random. Each group had 125 participants. The index knee among those with incident accelerated knee osteoarthritis or common knee osteoarthritis was the knee that first met the definition of incident accelerated knee osteoarthritis or common knee osteoarthritis, respectively. We defined the index knee of an individual with no knee osteoarthritis to be the same knee as that person’s matched member of the incident accelerated knee osteoarthritis group.

### Knee radiographs and semi-quantitative grading

Bilateral weight-bearing, fixed-flexion posteroanterior knee radiographs were obtained at baseline and the first 4 annual follow-up visits. Central readers, who were blinded to the order of follow-up radiographs, scored the images for KL grades (0 to 4). The agreement for these readings (read–reread) was good (weighted k [intrarater reliability] = 0.70–0.80). These KL grades are publicly available (files: kXR_SQ_BU##_SAS [versions 0.6, 1.6, 3.5, 5.5, and 6.3]) [6].

### Baseline coronal tibial slope

**Fig. 2:**
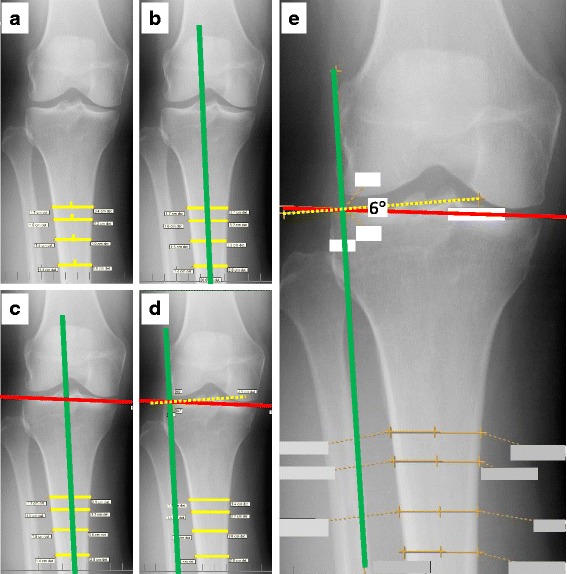
Example of the coronal tibial slope measurement. **a** Yellow lines are the medial-lateral lines with the midpoint of the diaphysis marked. **b** We added the longitudinal axis of the tibia (*green line*). **c** The red (*solid bold*) line connects the medial and lateral aspects of the tibial plateau. **d** The yellow (*dotted*) line is perpendicular to the long axis of the tibia. **e** The 6° angle indicates that the lateral edge of the tibial plateau is more proximal than the medial edge

We adapted methods described by Hashemi et al [7] to measure the baseline coronal tibial slope on weight-bearing, fixed-flexion posteroanterior knee radiographs. One reader (JBD) completed all of the measurements using EFilm Workstation 3.4 (Merge Healthcare, Chicago, IL, USA), which provides angular measurements as integers. First, we defined the longitudinal axis of the tibia by drawing at least four lines across the width of the diaphysis. The lines were parallel to the bottom of the radiograph (see Fig. 2a). In rare situations, where the radiographs lacked sufficient view of the tibial diaphysis, the reader placed one or two lines on the tibial metaphysis. After drawing the medial-lateral lines across the tibia, the reader marked the medial-lateral midpoint of the tibial diaphysis on each line (see Fig. 2a). The longitudinal axis of the tibia was created by drawing a line that passed through the medial-lateral midpoints of the tibia (see Fig. 2b). No knees had tibial bowing that prevented this method from detecting the longitudinal axis of the tibia. Next, the reader drew a line joining the peak points of the medial and lateral aspects of the tibial plateau (see Fig. 2c). The reader then shifted the longitudinal axis to the peak lateral aspect of the tibial plateau and drew a line perpendicular to the longitudinal axis (see Fig. 2d). If the peak lateral aspect was proximal to the peak medial aspect then the coronal tibial slope angle was positive (see Fig. 1). In contrast, if the peak lateral aspect was distal to the peak medial aspect then the coronal tibial slope angle was negative. The intra-reader reliability was good (ICC_3,1_ = 0.87, *n* = 15 knees) [8].

### Baseline femorotibial angle

Baseline femorotibial angle was measured on weight-bearing, fixed-flexion posteroanterior knee radiographs. These methods have been previously described [9] and the protocol and data are publicly available on the OAI website (file: kXR_FTA_Duryea00, version 0.2) [6]. To ensure an optimal sample size an additional 102 knees were measured by the same group that provided the original OAI measurements. In brief, a customized software tool, which was originally designed to measure location-specific joint space width, was adapted to quantify femorotibial angle. The program defined the tibial axis based on the central point of the knee and the center of the tibial shaft, typically 10 cm distal to the tibial plateau. The femoral axis was perpendicular to a line tangent to the distal ends of the medial and lateral femoral condyles. Valgus malalignment had more positive values than knees with varus malalignment. To classify knees with malalignment we adjusted femorotibial angles to match hip-knee-ankle angles as described in Iranpour-Boroujeni et al [9]. The intra- and inter-reader reliability was excellent (intra-reader ICC = 0.98 and inter-reader ICC = 0.98 and 0.99) [9].

### Clinical data (potential covariates or mediators)

Demographic, anthropometric, and other characteristics, which we selected a priori as potential covariates, were acquired based on a standard protocol. The data and protocol are publicly available [6]. We extracted several baseline variables to provide descriptive characteristics of the study sample or for analyses: age, body mass index, and self-reported knee injury [3]. At each follow-up visit participants were asked “Since your last annual visit to the OAI clinic about 12 months ago, have you injured your right knee badly enough to limit your ability to walk for at least two days?”. The staff asked a similar question for the left knee.

### Statistical analysis

To assess the relationship between coronal tibial slope (predictor) and incident accelerated knee osteoarthritis or common knee osteoarthritis (outcomes) compared with no knee osteoarthritis (reference outcome) we performed multinomial logistic regression analyses adjusted for sex (matching factor). We also adjusted for self-reported injury during the observation period (a possible mediator). We explored baseline body mass index as a possible confounder but we excluded it in the analyses because it was only weakly associated with coronal slope (*r* = 0.14) and changed the odds ratio < 10 %. We did not consider age a confounder because it is unlikely to influence the coronal tibial slope. Prior to conducting the primary analyses, we confirmed that coronal tibial slope had a linear relationship with the log odds for incident accelerated knee osteoarthritis and common knee osteoarthritis. We also explored stratified analyses among those with varus malalignment ( −2 degrees or less), valgus malalignment (2° or more), and normal alignment based on the adjusted femorotibial angles [9]. Finally, we performed a *post hoc* analysis with two logistic regression models to determine if the coronal slope was associated with the incidence medial or lateral joint space narrowing (outcomes). Results are reported as means (standard deviation) and odds ratio (OR) with 95 % confidence intervals (95 % CI). We conducted all analyses in SAS 9.4 (Cary, NC, USA) and defined statistical significance based on *p* ≤ 0.05.

## Results

We had complete data among matched individuals for 109 (87 %) adults per group. Table 1 has the descriptive baseline characteristics of each group. Coronal slope ranged from -3.0° to 9.0° and was inversely related to femorotibial angle (*r* = 0.47, *p* < 0.01; e.g., a slope with a more proximal lateral plateau was related with a greater varus malalignment). Baseline coronal tibial slope was not statistically different between those who had a recent injury (*n* = 66, slope = 2.9° (2.0)) or no injury (*n* = 258, slope = 2.8° (2.0), t = −0.54, *p* = 0.59).

**Table 1:** Baseline Characteristics of Study Sample

Variable	No Knee OA *n = 109*	Common Knee OA *n = 109*	Incident Accelerated Knee OA *n = 109*
Female, n (%)	70 (64 %)	70 (64 %)	70 (64 %)
Age (years), mean (SD)	58 (8)	58 (8)	63 (9)
BMI (kg/m2), mean (SD)	27.1 (4.5)	28.2 (4.5)	29.6 (4.5)
Femorotibial Angle (degrees), mean (SD)	−5.4 (1.7)	−5.2 (1.7)	−5.3 (1.6)

Notes: *OA* osteoarthritis, *BMI* body mass index, *SD* standard deviation

A greater coronal slope was associated with an increased risk of incident accelerated knee osteoarthritis (OR = 1.15, 95 % CI = 1.01 to 1.32) but not common knee osteoarthritis (OR = 1.04, 95 % CI = 0.91 to 1.19) compared with those without knee osteoarthritis (Table 2). Hence, for every degree increase in coronal slope, the odds of developing accelerated knee osteoarthritis increased by 15 %. These findings were similar when adjusted for recent injury: incident accelerated knee osteoarthritis OR = 1.16, 95 % CI = 1.01 to 1.33, common knee osteoarthritis OR = 1.04, 95 % CI = 0.91 to 1.19. Among knees with varus malalignment a greater coronal slope increased the odds of incident accelerated knee osteoarthritis; there was no significant relationship between slope and incident accelerated knee osteoarthritis among knees with normal alignment (see Table 2).

**Table 2:** Association between baseline tibial coronal slope and common knee osteoarthritis (KOA) or incident accelerated knee osteoarthritis (AKOA)

	No KOA (REF)	KOA	AKOA	Adjusted OR^a^ (per degree) KOA	Adjusted OR^a^ (per degree) AKOA
Full Sample (n = 327)	*n* = 109	*n* = 109	*n* = 109		
Baseline Tibial Coronal Slope Angle (deg, mean (SD))	2.6 (1.9)	2.7 (2.1)	3.1 (2.0)	1.04 (0.91, 1.19)	1.15 (1.01, 1.32)
Varus (*n* = 88)	*n* = 29	*n* = 26	*n* = 33		
Baseline Tibial Coronal Slope Angle (deg, mean (SD))	3.3 (1.5)	4.0 (2.3)	4.3 (1.4)	1.27 (0.92, 1.75)	1.38 (1.01, 1.88)
Valgus (*n* = 18)	*n* = 7	*n* = 7	*n* = 4		
Baseline Tibial Coronal Slope Angle (deg, mean (SD))	0.7 (2.0)	0.1 (1.3)	3.3 (3.1)	0.63 (0.28, 1.42)	1.59 (0.72, 3.53)
Neutral (*n* = 221)	*n* = 73	*n* = 76	*n* = 72		
Baseline Tibial Coronal Slope Angle (deg, mean (SD))	2.5 (1.9)	2.5 (1.8)	2.6 (1.9)	1.01 (0.85, 1.20)	0.99 (0.83, 1.19)

Notes: *OR* odds ratio, *KOA* knee osteoarthritis, *deg* degrees

^a^All models adjusted for sex (matching factor)

To understand the relationship between coronal slope and incident joint space narrowing we conducted two *post hoc* analyses among the 318 (97 %) participants who had joint space narrowing scores at baseline and 48-month visit. Within this sample, 102 adults had incident medial joint space narrowing (including 77 people with accelerated knee osteoarthritis). Furthermore, 35 individuals had incident lateral joint space narrowing (including 29 people with accelerated knee osteoarthritis). We found that a greater coronal slope was associated with increased odds of incident medial joint space narrowing (OR = 1.21, 95 % CI = 1.07 to 1.37) but not incident lateral joint space narrowing (OR = 1.03, 95 % CI = 0.87 to 1.23).

## Discussion

This is the first study to demonstrate that a greater coronal tibial slope is associated with incident accelerated knee osteoarthritis but not common knee osteoarthritis. Hence, the more proximal the lateral tibial plateau margin relative to the medial tibial plateau, the greater the risk for incident accelerated knee osteoarthritis (e.g., Fig. 1b), particularly among knees with malalignment. Based on *post hoc* analyses, coronal tibial slope may primarily relate with incident medial joint space narrowing and not lateral joint space narrowing.

Coronal tibial slope is associated with altered joint biomechanics during a landing task [5]. A decreased coronal tibial slope is associated with increased knee valgus angles during landing, while an increased coronal tibial slope is associated with greater knee internal rotation excursions [5]. While landing activities are not common among older adults these findings highlight that the coronal tibial slope could influence joint biomechanics. It will be interesting to confirm if coronal tibial slope angle influences joint biomechanics during more common tasks (e.g., walking, stair climbing), particularly among those with knee malalignment. Contrary to our hypothesis, we found a linear relationship indicating that a greater tibial slope increases the odds of developing accelerated knee osteoarthritis. The lack of a U-shaped relationship, in which any deviation of coronal tibial slope is associated with incident accelerated knee osteoarthritis, may be explained by future research that explores the relationship between coronal tibial slope and gait mechanics. Furthermore, future biomechanical studies may clarify why the coronal tibial slope is associated with medial joint space narrowing but not lateral joint space narrowing. One possibility is that the geometry of the medial tibiofemoral compartment causes the compartment to be more sensitive to subtle changes in joint geometry [10]. These future studies also need to explore how coronal tibial slope and knee malalignment interact to influence gait mechanics.

The coronal tibial slope may be associated with incident accelerated knee osteoarthritis among individuals with knee malalignment but not among adults with normal static knee alignment. Hence, static knee alignment may moderate the relationship between coronal tibial slope and incident accelerated knee osteoarthritis. Knees with normal alignment may tolerate deviations in the coronal slope. In contrast, knees with static malalignment may be more sensitive to small perturbations in the coronal tibial slope. This suggests that the coronal tibial slope may provide additional information not captured by static knee malalignment. Static knee alignment relies on the longitudinal axes of the femur and tibia while the coronal tibial slope is defined by the angle of the tibial plateau relative to the longitudinal axis of the tibia. If static knee alignment and coronal tibial slope represented similar constructs we would have expected correlations greater than *r* = 0.47. We believe it may be valuable for researchers to measure both the coronal tibial slope when they assess static knee alignment. Furthermore, future work should explore if the coronal tibial slope influences the effects of biomechanical interventions for knee osteoarthritis.

We rejected our hypothesis that a recent knee injury was a mediator in the relationship between coronal tibial slope and incident accelerated knee osteoarthritis. We found no differences in coronal tibial slope between those who had a knee injury during the observation period and those who did not. Furthermore, adjusting for a recent knee injury had no influence on the association between greater coronal tibial slope and increased accelerated knee osteoarthritis risk. These results are supported by a prior study, which also found no association between coronal tibial slope and injury risk [11].

While these findings are novel, it is important to note that the sample size limited our ability to explore if coronal tibial slope interacts with combinations of other factors that alter joint loading. For example, an obese individual with malalignment and a greater tibial slope may be at greater risk for incident accelerated knee osteoarthritis than someone with only one or two of these risk factors. Furthermore, we could not confirm if the coronal tibial slope is an independent predictor of incident accelerated knee osteoarthritis. Despite this limitation, our exploratory analyses indicated that an individual with malalignment and a greater coronal tibial slope is at greater risk for incident accelerated knee osteoarthritis. A second limitation is that the OAI only collected self-reported injury and the type and severity of the knee injury remains unknown. Future studies should explore whether certain types of injuries or greater severity of injuries may mediate the relationship between coronal tibial slope and incident accelerated knee osteoarthritis.

## Conclusions

In conclusion, coronal tibial slope, particularly among knees with malalignment, may be an important risk factor for incident accelerated knee osteoarthritis, which is a painful and disabling type of osteoarthritis [4]. The slope measure may be a risk factor unique to incident accelerated knee osteoarthritis that may eventually help identify individuals at risk for incident accelerated knee osteoarthritis, particularly in the medial tibiofemoral compartment. This builds on the existing literature that older age, greater body mass index, new knee injury, and coronal tibial slope may be important risk factors for incident accelerated knee osteoarthritis. Future research should account for coronal tibial slope and explore if coronal tibial slope interacts with combinations of other factors that alter joint loading (e.g., obesity with malalignment) to increase the risk of incident accelerated knee osteoarthritis.

## Abbreviations

CI, confidence interval; ICC, intraclass correlation; KL, Kellgren-Lawrence; OAI, osteoarthritis initiative; OR, odds ratio
